# Intralipid Decreases Apolipoprotein M Levels and Insulin Sensitivity in Rats

**DOI:** 10.1371/journal.pone.0105681

**Published:** 2014-08-21

**Authors:** Lu Zheng, Yuehua Feng, Yuanping Shi, Jun Zhang, Qinfeng Mu, Li Qin, Maria Berggren-Söderlund, Peter Nilsson-Ehle, Xiaoying Zhang, Guanghua Luo, Ning Xu

**Affiliations:** 1 Comprehensive Laboratory, the Third Affiliated Hospital of Soochow University, Changzhou, P.R. China; 2 Department of Cardiothoracic Surgery, the Third Affiliated Hospital of Soochow University, Changzhou, P.R. China; 3 Division of Clinical Chemistry and Pharmacology, Department of Laboratory Medicine, Lunds University, Lund, Sweden; Virgen Macarena University Hospital, School of Medicine, University of Seville, Spain

## Abstract

**Background:**

Apolipoprotein M (ApoM) is a constituent of high-density lipoproteins (HDL). It plays a crucial role in HDL-mediated reverse cholesterol transport. Insulin resistance is associated with decreased ApoM levels.

**Aims:**

To assess the effects of increased free fatty acids (FFAs) levels after short-term Intralipid infusion on insulin sensitivity and hepatic ApoM gene expression.

**Methods:**

Adult male Sprague-Dawley (SD) rats infused with 20% Intralipid solution for 6 h. Glucose infusion rates (GIR) were determined by hyperinsulinemic-euglycemic clamp during Intralipid infusion and plasma FFA levels were measured by colorimetry. Rats were sacrificed after Intralipid treatment and livers were sampled. Human embryonic kidney 293T cells were transfected with a lentivirus mediated human apoM overexpression system. Goto-Kakizaki (GK) rats were injected with the lentiviral vector and insulin tolerance was assessed. Gene expression was assessed by real-time RT-PCR and PCR array.

**Results:**

Intralipid increased FFAs by 17.6 folds and GIR was decreased by 27.1% compared to the control group. ApoM gene expression was decreased by 40.4% after Intralipid infusion. PPAR_β/δ_ expression was not changed by Intralipid. Whereas the mRNA levels of Acaca, Acox1, Akt1, V-raf murine sarcoma 3611 viral oncogene homolog, G6pc, Irs2, Ldlr, Map2k1, pyruvate kinase and RBC were significantly increased in rat liver after Intralipid infusion. The Mitogen-activated protein kinase 8 (MAPK8) was significantly down-regulated in 293T cells overexpressing ApoM. Overexpression of human ApoM in GK rats could enhance the glucose-lowering effect of exogenous insulin.

**Conclusion:**

These results suggest that Intralipid could decrease hepatic ApoM levels. ApoM overexpression may have a potential role in improving insulin resistance *in vivo* and modulating apoM expression might be a future therapeutic strategy against insulin resistance in type 2 diabetes.

## Introduction

Apolipoprotein M (ApoM) is a constituent of plasma high-density lipoproteins (HDL) and most plasma ApoM are bound to HDL, which plays an important role on lipid and lipoprotein metabolism [1], [2]. ApoM could influence pre-β HDL formation and cholesterol efflux, which is thought to be one of key regulators of HDL metabolism and reverse cholesterol transport [1], [2]. It has been demonstrated that ApoM expression could be directly regulated by the hepatic nuclear factor-1α (HNF-1α) [3], liver receptor homolog-1 (LRH-1) [4], forkhead box A2 (Foxa2) [5], and liver X receptor (LXR) [5]. And all of these transcription factors are also involved in hepatic lipid and glucose metabolism [3]–[6].

Type 2 diabetes is a major health problem and its prevalence increased dramatically in the last decades, mostly due to obesity and sedentary lifestyle [7], [8]. Furthermore, insulin resistance, a key feature of type 2 diabetes, induces major metabolic abnormalities, resulting in high free fatty acids (FFA) plasma levels, hypertriglyceridemia, low HDL levels and small dense LDL particles [9], [10]. In addition, size and composition of HDL particles are abnormal in diabetic patients [11]. Indeed, serum/plasma ApoM levels are significantly reduced in diabetic and metabolic syndrome patients [12]–[14].

Since insulin resistance is one of the key features of type 2 diabetes, finding new ways to improve insulin resistance is important for the management of these patients. In vitro insulin and insulin-like growth factor I (IGF-I) could significantly inhibit apoM expression with a dose- and time-dependent manner [15], [16]. Moreover, both *in vivo* and *in vitro* observations suggested that ApoM may also be associated with diabetes and obesity [12]–[14]. Exogenous insulin administration could partially reverse abnormal ApoM expression in diabetic rats [17]. ApoM levels were significantly decreased in hyperglycemic rats, and high glucose and insulin concentrations inhibited ApoM expression in cultured cells [16].

Intralipid is a solution of soybean oil, phosphatidylcholine, glycerol and water, and is used to increase FFA levels. It contains significant amounts of ω-6 polyunsaturated fatty acids (PUFA) that are easily oxidized to generate reactive oxygen species [18]. Short-term Intralipid infusion significantly increases FFA levels and insulin resistance [19], [20] by decreasing peripheral glucose uptake [21] and down-regulating intracellular insulin signaling [22], [23]. Elevated FFA levels decrease insulin sensitivity in trained and sedentary humans [24], and induce insulin resistance in both skeletal and cardiac musles [25]. FFAs are ligands for ApoM in plasma, which could contribute to FFA removal from the circulation, preventing their ill effects [26].

We hypothesized that downregulation of ApoM expression by hyperglycemia may be associated with insulin resistance. In the present study, we studied the effects of artificially increasing FFAs on ApoM expression and insulin sensitivity in rats. We showed that increased FFA levels decreased both ApoM levels and insulin sensitivity. Therefore, modulating ApoM expression might be a future therapeutic strategy against insulin resistance in type 2 diabetes.

## Materials and Methods

### Animals

Each experimental group contained 5–6 adult male Sprague-Dawley (SD) rats (286.2±18.3 g) or, as a model for insulin resistance, aged male Goto-Kakizaki (GK) rats (416.1±40.0 g). In the present study, 10 male SD rats (8 weeks old) underwent a hyperinsulinemic-euglycemic clamp (HEC) and 10 aged male GK rats (32 weeks old) were obtained from the Shanghai Slac Laboratory Animal Co., China. Another 12 male SD rats (8 weeks old) were obtained from the Changzhou Cavens Laboratory Animal Co., China.

Rats were kept in a temperature-controlled (22°C) room with 12-hrs light-dark cycle, and were provided with standard rodent chow and water ad libitum. Rats were acclimatized for one week before placing catheters. All procedures and animal experiments were approved by the Animal Care and Use Committee of the Soochow University (Suzhou, China) (permit number SYXK(Su)2002–0045)).

### Surgical preparation

After being acclimated to their new environment, SD rats underwent surgery to place catheters 7 days before experiments. Rats were anesthetized with 10% chloral hydrate (4 ml/kg). Two catheters were placed, one in each jugular vein: one for Intralipid infusion, and the other for 20% glucose infusion during hyperinsulinemic euglycemic clamp (HEC). An additional catheter was placed in a carotid artery for blood sampling. The free ends of the catheters were attached to steel tubing and tunneled subcutaneously on the back of the neck. The catheters were flushed with isotonic saline containing 50 IU/ml heparin (Qianhong Bio-pharma Co., Ltd., Changzhou, China) and filled with a viscous solution of heparin (500 IU/ml) and 300 g/L polyvinyl pyrolidone (PVP-10; Sigma, St Louis, MO, USA) to prevent blood reflux into the catheter lumen.

### Intralipid infusion

20% Intralipid (Sino-Swed Pharmaceutical Corp, Ltd., Jiangsu, China) containing 20% soybean oil, 1.2% lecithin and 2.2% glycerin, is a triglyceride emulsion, which releases fatty acids (Table 1) with the concomitant infusion of heparin, a stimulant of the lipoprotein lipase enzyme. On the day of the experiment, the catheters were carefully connected to infusion pumps (Smiths Medical, Lower Pemberton, UK). A 20% Intralipid solution (10 ml·kg^−1^·h^−1^) combined with heparin (0.0975 IU/min) was infused for 6 h. In addition, Intralipid/heparin was infused via the tail vein in another parallel experiments, without HEC test, to avoid the interference of the 20% glucose infusion on liver gene expression. All control rats received 5% glucose solution (10 ml·kg^−1^·h^−1^) combined with heparin (0.0975 IU/min). After Intralipid treatment for 6 h, SD rats were anesthetized using 10% chloral hydrate (4 ml/kg) and sacrificed. Blood samples were obtained from the inferior vena cava. Plasma was separated by centrifugation and stored at −70°C. Livers were removed, sectioned, and stored in liquid nitrogen.

**Table 1:** Fatty acid composition of soybean oil in 20% Intralipid.

Fatty acid	Ratio (% w/w)
<C14∶0	≤0.1
Myristic acid	≤0.2
Palmitic acid	9.0–13.0
Palmitoleic Acid	≤0.3
Stearic acid	3.5–5.0
Oleic acid	17.0–30.0
Linoleic acid	48.0–58.0
Linolenic acid	5.0–11.0
Arachidic acid	≤0.1
Eicosenoic acid	≤0.1
Docosanoic acid	≤0.1

### Hyperinsulinemic-euglycemic clamp

To assess insulin sensitivity, rats underwent a primed-constant infusion of 10 mU/kg/min of insulin to achieve a steady state. A 20% D-glucose solution (AMRESCO Inc., Solon, OH, USA) was then infused. Blood glucose was measured at 5-min intervals using an ACCU-CHECK Active glucosimeter (Roche Diagnostics, Basel, Switzerland). The glucose infusion rate (GIR) was adjusted in order to maintain a blood glucose level of approximately 5.5 mmol/L. The mean glucose infusion rate in the last 30 min was used for analysis [27].

### Determinations of FFAs

FFAs were determined with the nonesterified fatty acid (NEFA) colorimetric method (Applygen Technologies Inc, Beijing, China). Briefly, total plasma FFAs were extracted with a chloroform:N-heptane:methanol solution (56∶42∶2), coupled with copper, reacted with color reagent and measured with a UV-2401PC UV-visible spectrophotometer (Shimadzu, Tokyo, Japan) at 550 nm. The standard curve was created using a series of dilution of palmitic acid.

### Lentiviral expression system for overexpression of human ApoM gene

A lentiviral expression system for overexpression of the human ApoM gene was constructed by Shanghai GenePharma Co., Ltd (Shanghai, China). A 564-bp fragment of the ApoM gene (GenBank accession number: AF118393) was cloned into a lentivirus transfection vector. In brief, a plasmid containing the ApoM gene, a plasmid encoding the Gag/Pol gene, a plasmid encoding the rev gene, and a plasmid encoding the vesicular stomatitis virus G glycoprotein gene were co-transferred into human embryonic kidney 293T cells (HEK 293T) using lipofectamine 2000 (Invitrogen Inc., Carlsbad, CA, USA). A control lentivirus vector expressing green fluorescent protein (GFP) was constructed in the same manner, but without the ApoM gene.

HEK 293T cells (ATCC, Manassas, VA, USA) were cultured in RPMI1640 supplemented with 10% fetal bovine serum (GIBCO, Invitrogen Inc., Carlsbad, CA, USA), 100 U/ml of penicillin, 100 µg/ml of streptomycin and 2 mM of L-glutamine (Invitrogen, Carlsbad, CA, USA) at 37°C in a 5% CO_2_ humidified incubator. Cells were seeded in 6-well cell culture plates, and were grown to 50–70% confluence. Prior to experiments, cells were washed once with phosphate buffered saline (PBS), and once with serum-free RPMI1640 without antibiotics. In each well, the experimental medium contained 3 ml of RPMI1640 with 1.0% human serum albumin (HSA), 50 µl of 1×10^9^ TU lentivirus expressing GFP and ApoM simultaneously (n = 6) or lentivirus only expressing GFP (control group, n = 6), and 15 µg of polybrene (Invitrogen, Carlsbad, CA, USA).

### Insulin tolerance test

An insulin tolerance test (ITT) was performed in GK rats (n = 5 for each group) 14 days after being transfected with 5×10^8^ TU of lentiviral vectors with or without the human ApoM gene via tail vein injection. After a 5-h fast, rats were injected with 1 IU/kg of insulin (Wanbang Biopharmaceuticals, China) intra-peritoneally. Blood was sampled from the tail vein 0, 30, 60, 90, 120, 150, 180 and 210 min after insulin injection. Blood glucose levels were determined using a glucosimeter (ACCU-CHEK Active, Roche Diagnostics, Basel, Switzerland). Blood glucose half-time (t1/2) was calculated from the slope of the least squares regression line of the blood glucose concentration during the linear phase of decrease.

After ITT, GK rats were anesthesized using 10% chloral hydrate (4 ml/kg) and sacrificed. Lungs, livers and kidneys were removed, sectioned, and stored in liquid nitrogen.

### Real-time Reverse transcription (RT)-PCR and PCR array

Total RNA from 293T cells, liver tissues from SD rats and liver, kidney and lung tissues from GK rats was extracted using a total RNA purification kit (Omega Bio-Tek Inc., Norcross, GA, USA), according to the manufacturer's instructions. RNA quality was determined by absorbance at 260/280 nm. Using the first strand cDNA synthesis kit (Qiagen, Venlo, Netherlands), 2 µg of total RNA was reverse transcribed to cDNA. PCR primers were designed according to the data available in GenBank (Table 2). β-actin was used as the reference gene. Relative standard curves were created to compensate for PCR efficiency. mRNA levels are expressed in relation to β-actin mRNA level. The real-time PCR reaction for each gene was performed in a 25 µL volume, in a glass capillary containing 0.4 µM of each primer and probe, 2 µL of cDNA, 1× buffer, 1.5 mM of MgCl_2_, 200 µM of dNTPs, and 1.25 U of Taq DNA polymerase. Thermal cycling conditions were: initial denaturation at 95°C for 3 min, followed by 40 cycles at 95°C for 5 sec and 60°C for 15 sec for Rat PPARβ/δ (rat ApoM, 58°C for 12 sec; rat β-actin, 61°C for 10 sec). All PCRs were performed on a LightCycler real-time PCR system (Roche Diagnostics, Basel, Switzerland). The human ApoM gene quantification assay was performed according to a published method [28].

**Table 2:** Sequences of primers and probes.

Gene	Primer/Probe	Sequence (5′-3′)	Product (bp)
Rat ApoM	Forward	acaaagagaccccagagccc	67
	Reverse	tccatggtgggagccg	
	Probe	FAM-acctgggcctgtggtactttattgctgg-TAMRA	
Rat β-actin	Forward	gccactgccgcatcctct	108
	Reverse	ctggaagagagcctcgggg	
	Probe	FAM-agctgcctgacggtcaggtcatcactatc-TAMRA	
Rat PPARβ/δ	Forward	gaagaaccgcaacaagtgtcagta	85
	Reverse	ccttccaaagcggatagcgt	
	Probe	FAM-cttccagaagtgcctggcgctcggc-TAMRA	

To scan genes of interest in liver of SD rats administrated with 20% Intralipid solution and in 293T cells transfected with a lentivirus mediated human apoM overexpression system, we used the PCR Array analyses for genes related to Rat Insulin Signaling Pathway (PARN-030Z) (SABiosciences, Qiagen, Venlo, Netherlands) in rats, and Human Type 2 Diabetes Mellitus (PA2) (CT bioscience, Jiangsu, China) in 293T cells, according to the manufacturer's instructions. PCR array data were calculated by the comparative cycle threshold method, normalized against multiple housekeeping genes, and expressed as mean fold change in experimental samples relative to control samples.

### Statistical analysis

Data are expressed as means ± standard error of the mean (SEM). Statistical analyses were performed using GraphPad Prism 6.0 (GraphPad Software, Inc., San Diego, California, USA). Comparisons between two groups were evaluated by unpaired *t*-tests. *P*-values <0.05 were considered significant.

## Results

### Intralipid decreased insulin sensitivity and inhibited ApoM gene expression

**Figure 1:**
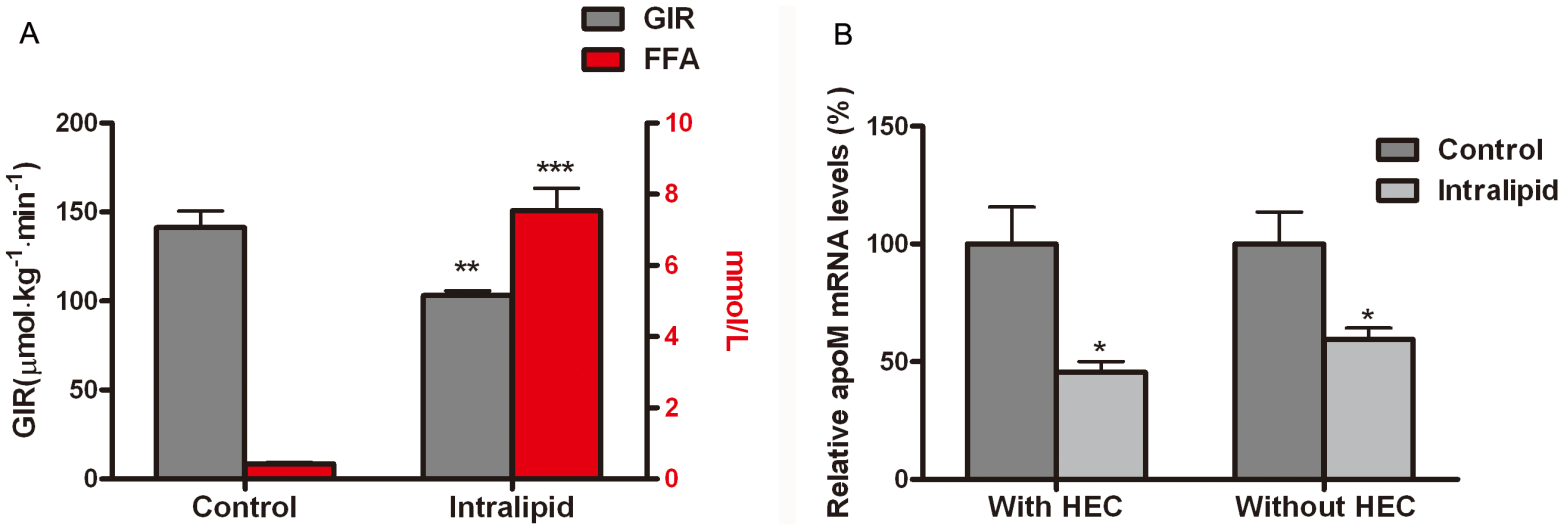
Effects of Intralipid on insulin sensitivity, free fatty acids (FFA) levels and hepatic ApoM gene expression in SD rats. (A) Plasma FFA levels in rats after infusion of 5% glucose solution (controls, n = 6) or 20% Intralipid solution (n = 6) for 6 h, and glucose infusion rate (GIR) during hyperinsulinemic euglycemic clamp (HEC) in control rats (n = 5) and Intralipid-infused rats (n = 5). (B) Hepatic ApoM mRNA levels in control rats and in Intralipid-infused rats with (n = 5 for each group) or without (n = 6 for each group) HEC were determined by real-time RT-PCR. ApoM mRNA levels in control rats were set as 100%. Data are presented as means ± standard error of the mean (SEM). *P<0.05, **P<0.01, ***P<0.001 *vs.* controls.

In SD rats infused with 20% Intralipid, plasma FFA levels increased by 17.6-folds and GIR was reduced by 27.1% compared with SD rats infused with 5% glucose solution (both *P*<0.01) (Figure 1A). As shown in Figure 1B, liver ApoM gene expression was significantly inhibited by 40.4% after Intralipid infusion followed by HEC, compared with control SD rats. When the HEC test was omitted to avoid the potentially confounding effect of 20% glucose, ApoM mRNA levels were still significantly decreased by 40.4% in SD rats infused with Intralipid compared with control SD rats (Figure 1B). There was no significant difference in ApoM mRNA levels between rats infused with Intralipid with or without HEC (*P*>0.05).

### Effects of short-term Intralipid infusion on genes related to the insulin signaling pathway in rat liver

**Figure 2:**
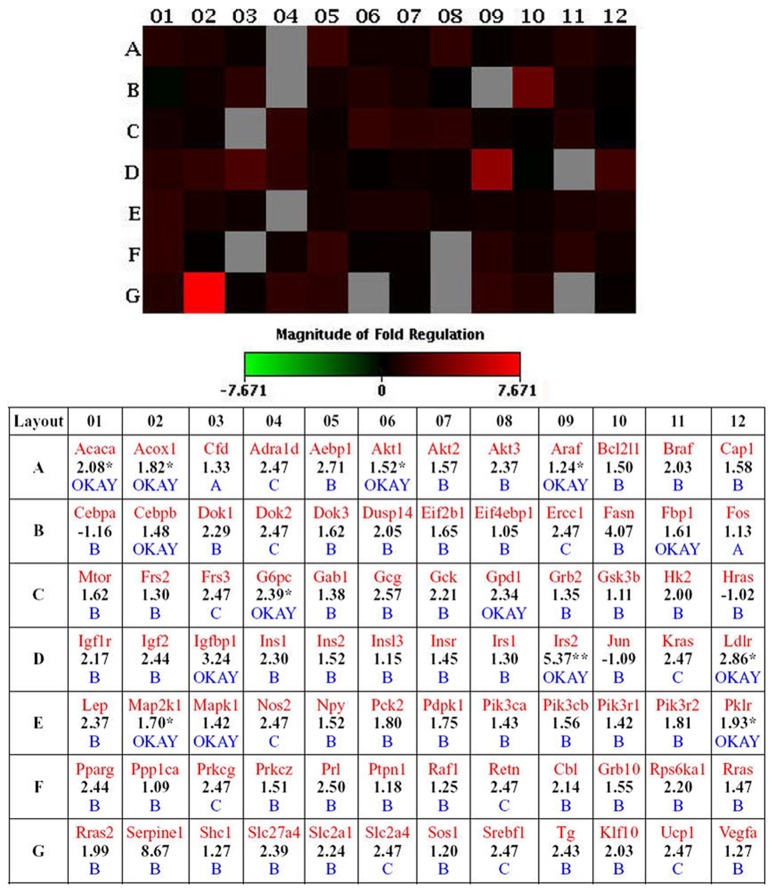
Effects of Intralipid on genes related to insulin signaling in liver tissues of SD rats. Genes related to insulin signaling were determined by PCR array. Fold changes of certain liver genes in Intralipid-infused SD rats (experimental group) compared with 5% glucose solution (control group). *P<0.05 and **P<0.01 *vs.* control group; (n = 6 for each group, total of 12 PCR arrays, all genes normalized to β-actin individually). Numbers in bold represent fold-changes. Genes are identified using their abbreviated name (in red). A (in blue): Average threshold cycle was relatively high (>30) in the control or the experimental sample, and was reasonably low in the other sample (<30). B (in blue): Average threshold cycle was relatively high (>30), meaning that its relative expression level was low, in both control and experimental samples, and the *P*-value for the fold-change was either unavailable or high (*P*>0.05). C (in blue): Average threshold cycle was either not determined or greater than the predefined cut-off value (default value of 35) in both samples, meaning that its expression was undetected, making the result erroneous and non-interpretable. “OKAY” indicates that threshold cycle in the control and the experimental sample was accepted (<30).

Using PCR array, we examined 84 genes reportedly related to the insulin response in SD rats. We observed that the mRNA levels of acetyl-CoA carboxylase alpha (Acaca), acyl-CoA oxidase 1 (Acox1), v-akt murine thymoma viral oncogene homolog 1 (Akt1), V-raf murine sarcoma 3611 viral oncogene homolog (Araf, component of MAPK pathway), catalytic subunit (G6pc), insulin receptor substrate 2 (Irs2), low density lipoprotein receptor (Ldlr), mitogen-activated protein kinase kinase 1 (Map2k1) and pyruvate kinase in liver and RBC (Pklr, target gene for SREBP1) were significantly increased in SD rats treated with Intralipid (Figure 2).

### Effect of Intralipid on PPAR_β/δ_ mRNA expression in rat liver

**Figure 3:**
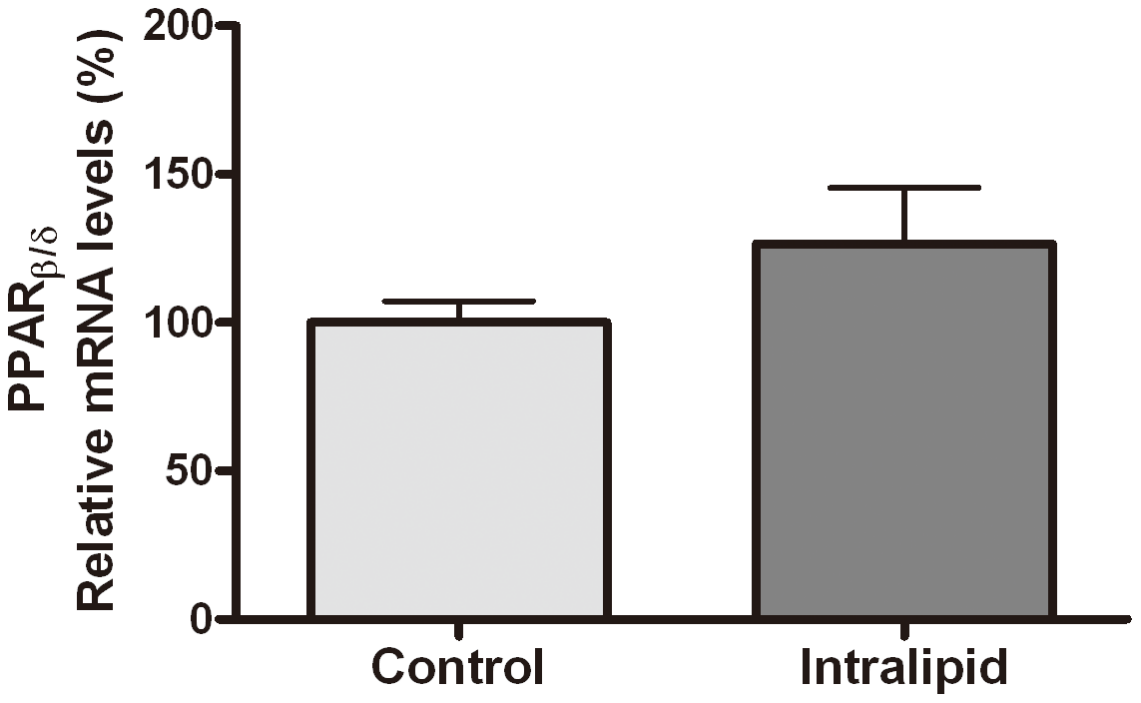
Effects of Intralipid on hepatic PPAR_β/δ_ mRNA expression in SD rats. Total RNA was obtained from the liver of rats after infusion of 5% glucose solution (controls, n = 6) or rats infused with 20% Intralipid solution (n = 6). mRNA expression was determined by real-time PCR. mRNA levels in control rats were set as 100%. Data are presented as means ± SEM.

We previously reported [15] that activation of PPAR_β/δ_, but not PPAR_α_ and PPAR_γ_, might be involved in the down-regulation of ApoM. Since the gene chip did not contain the PPAR_β/δ_ gene, we detected the mRNA levels of PPAR_β/δ_ by real-time RT-PCR after infusion of 20% Intralipid in SD rats. Intralipid soluton did not significantly influence the hepatic PPAR_β/δ_ mRNA expression in rats (*P* = 0.21) (Figure 3).

### Overexpression of human ApoM gene on insulin sensitivity in 293T cells

**Figure 4:**
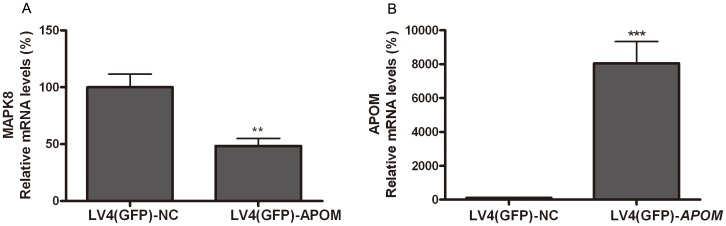
Human ApoM overexpression increased MAPK8 mRNA expression in 293T cells. Lentiviral vectors (LV) were used as delivery vehicles. 293T cells were treated with LV4(GFP)-NC that expressed GFP alone (control group, n = 6) or LV4(GFP)-ApoM that expressed GFP and ApoM (n = 6). (A) MAPK8 mRNA levels. (B) ApoM mRNA levels. mRNA expression was determined by real-time RT-PCR. mRNA levels in control rats were set as 100%. Data are presented as means ± SEM. **P<0.01 and ***P<0.001 *vs.* controls.

PCR array analyses using the Human Type 2 Diabetes Mellitus (PA2) array in 293T cells overexpressing human ApoM (Table S1) demonstrated that the mitogen-activated protein kinase 8 (MAPK8) gene, which is related to insulin resistance [29], was down-regulated by 2.1 folds (*P* = 0.0029) (Figure 4A), while ApoM mRNA levels in 293T cells transfected with the human ApoM gene were increased by 80 folds (*P* = 0.0001) (Figure 4B).

### Overexpression of human ApoM gene on insulin sensitivity in GK rats

**Figure 5:**
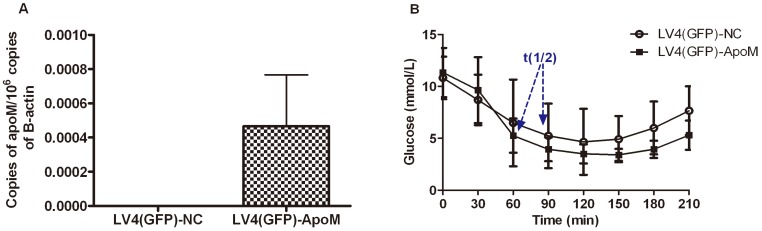
Human ApoM overexpression in Goto-Kakizaki (GK) rats improves insulin resistance. GK rats were injected with 5×10^8^ TU of LV4(GFP)-NC that expressed GFP alone (control group, n = 5) or LV4(GFP)-ApoM that expressed GFP and ApoM (n = 5) via the tail vein. (A) Human ApoM mRNA expressions in lungs of GK rats. mRNA expression was determined by real-time RT-PCR. Copy number of ApoM in the lungs of GK rats treated with the LV4(GFP)-NC or LV4(GFP)-ApoM, respectively. (B) Insulin tolerance test (ITT) was performed on day 14. Blood was sampled from the tail vein at 0, 30, 60, 90, 120, 150, 180 and 210 min after insulin administration, and blood glucose half-time (t1/2) was calculated from the slope of the least squares regression during the linear phase of decline. Data are presented as means ± SEM.

We investigated the effects of overexpressing the human ApoM gene on insulin sensitivity in GK rats, which is a non-obese Wistar substrain rat characterized by mild hyperglycemia, insulin resistance and hyperinsulinemia [30], [31]. As shown in Figure 5A, human ApoM mRNA levels were significantly increased in the lungs (No human ApoM gene expression in kidneys and livers) of GK rats after injecting 5×10^8^ TU of lentiviral vectors integrating the human ApoM gene. There was no expression of the human ApoM gene in the lungs of GK rats transfected with the LV4(GFP)-NC vector. After 14 days, there were no obvious differences in fasting blood glucose levels between control rats and rats transfected with the human ApoM gene (10.82±0.92 mmol/L *vs.* 11.34±1.06 mmol/L, *P* = 0.72). Interestingly, ITT analysis (Figure 5B) showed that the blood glucose t1/2 of GK rats transfected with the human ApoM gene (61.5 min) was shorter than in control rats (89.0 min), although the difference did not reach statistical significance (*P* = 0.15), which may be due to the limited number of animals.

## Discussion

The aim of the present study was to assess the effects of increased FFAs levels after short-term Intralipid infusion on insulin sensitivity and hepatic ApoM gene expression. Intralipid could increase FFAs by 17.6 folds and decrease GIR by 27.1%. This further confirms previously findings [32] that Intralipid infusion increases plasma FFA levels and simultaneously decreases insulin sensitivity.

ApoM is the acceptor of HDL-carrying S1P [33], apoM could enhance HDL-mediated anti-oxidation effects [34], and it plays an important role for the pre-β HDL formation [35]. In the present study, we demonstrated that ApoM expression was also associated with insulin sensitivity. Artificially increased FFA levels after short-term Intralipid infusion could decrease ApoM expression. In order to minimize the experiment bias, the rats in the control group received glucose to avoid the confounding effect of starving on normal physiological processes. As expected, infusion of 20% Intralipid solution resulted in elevated plasma FFAs levels and decreased insulin sensitivity [5], [32], [36], [37]. Intralipid infusion could also decrease hepatic apoM expression in rats. To explore the mechanisms of down-regulation of ApoM by FFA, we monitored the expression of 84 genes related to the insulin response in liver tissues on rats after Intralipid infusion. The mRNA levels of Acaca, Acox1, Akt1, Araf, G6pc, Irs2, Ldlr, Map2k1 and pyruvate kinase, which are all proteins involved in energy metabolism and insulin signaling in the liver, were all significantly increased. A number of transcription factors are involved in the liver response to increased FFA levels and in insulin signaling, such as HNF-1α, LRH-1, Foxa2, and LXR [38]-[40]. These transcriptions factors are also involved in ApoM transcription, suggesting that ApoM may directly or indirectly involved in energy source metabolism and insulin signaling [3]-[6].

We have previously reported [15] that activation of PPAR_β/δ_, but not PPARα and PPARγ, might be involved in the down-regulation of ApoM, suggesting that the FFA-induced down-regulation of ApoM expression may be mediated, at least in part, by the PPAR_β/δ_ pathway. However, in the present study, Intralipid infusion did not influence hepatic PPAR_β/δ_ mRNA expression, despite marked elevations in plasma FFA levels. This discrepancy may be explained by the fatty acid composition of Intralipid (linoleic acid 44–62%, oleic acid 19–30%, palmitic acid 7–14%, linolenic acid 4–11% and stearic acid 1.4–5.5%). The effects of different FFA on PPAR_β/δ_ expression warrant further studies.

Lentiviruses can permanently integrate genetic material into the genome of host cells, are able to maintain long-term expression of integrated material, and can be used to express transgenes and to suppress the expression of endogenous genes by RNA interference (RNAi) [41]. In the present study, we constructed lentiviral vectors to overexpress the human ApoM gene in order to increase ApoM expression in 293T cells and in GK rats. Our results demonstrated that MAPK8 was significantly down-regulated, while ApoM mRNA levels in 293T cells transfected with human ApoM gene were dramatically increased. MAPK8 is known as the c-Jun NH_2_-terminal kinase 1 (JNK1), and is involved in the mechanism of obesity-induced insulin resistance [29]. Feeding a high fat diet can cause activation of the JNK1 signaling pathway, insulin resistance, and obesity in mice [29]. Interestingly, ITT analysis showed that human ApoM expression in GK rats had a tendency to enhance the glucose-lowering effects of exogenous insulin, suggesting that overexpression of ApoM might improve insulin sensitivity. However, further studies are needed to confirm this observation and to clarify the mechanism.

In conclusion, the present study demonstrated that Intralipid could increase plasma FFA levels, decreased insulin sensitivity and suppressed ApoM expression. Moreover, Intralipid could enhance a number of genes involved in insulin signaling. Our results suggest that ApoM overexpression may have a potential role in improving insulin resistance in *vivo*, and could be considered as a future therapeutic target against insulin resistance and type 2 diabetes.

## Supporting Information

Table S1 (DOC)
