# Supplementary material for: Intralipid Decreases Apolipoprotein M Levels and Insulin Sensitivity in Rats
Source: PLoS One. 2014 Aug 21;9(8):e105681. doi: 10.1371/journal.pone.0105681 (PMC4140822; doi:10.1371/journal.pone.0105681)
Supplement: Table S1 — (DOC) [file pone.0105681.s001.doc]

**Table S1. Effects of ApoM overexpression on genes related to human type 2 diabetes mellitus** in 293T cells

| **Gene** | **Fold-change** | ***P*-value** |  | **Gene** | **Fold-change** | ***P*-value** |
| --- | --- | --- | --- | --- | --- | --- |
| AACS | -1.01 | 0.946 |  | IGFBP1 | 1.21 | 0.552 |
| ACVR1C | 1.07 | 0.727 |  | IKBKB | 1.05 | 0.813 |
| ACVR2B | 1.07 | 0.301 |  | IL10 | 1.42 | 0.534 |
| AKT1 | -1.03 | 0.816 |  | IL1B | -1.35 | 0.204 |
| AKT2 | -1.15 | 0.476 |  | IL2 | 1.23 | 0.598 |
| AKT3 | 1.05 | 0.600 |  | IL4 | -1.08 | 0.595 |
| CAMK2G | 1.09 | 0.463 |  | ILDR2 | -1.18 | 0.489 |
| CAPN10 | -1.05 | 0.808 |  | INHBB | 1.50 | 0.448 |
| CD38 | 1.02 | 0.926 |  | INS | -1.21 | 0.623 |
| CPE | 1.06 | 0.545 |  | INSR | 1.23 | 0.214 |
| CPLX3 | 1.06 | 0.811 |  | IRS2 | 1.04 | 0.259 |
| CPT1A | 1.12 | 0.621 |  | IRS4 | 1.02 | 0.937 |
| DGAT1 | 1.00 | 0.952 |  | JAK2 | 1.11 | 0.619 |
| DOK1 | -1.07 | 0.643 |  | LEP | 1.72 | 0.303 |
| DOK2 | -3.86 | 0.190 |  | MAFA | 1.19 | 0.324 |
| DOK3 | -1.45 | 0.060 |  | MAPK1 | -1.01 | 0.945 |
| DOK4 | -1.11 | 0.526 |  | MAPK10 | -1.02 | 0.894 |
| DOK5 | 2.08 | 0.051 |  | MAPK3 | -1.02 | 0.835 |
| EIF2AK3 | -1.09 | 0.474 |  | MAPK8 | -2.06 | **0.003**** |
| EIF4EBP2 | 1.83 | 0.187 |  | MAPK9 | -1.04 | 0.798 |
| ENPP1 | -1.03 | 0.789 |  | NOS2 | -1.13 | 0.525 |
| ERBB3 | 1.05 | 0.760 |  | PDE3B | 1.09 | 0.793 |
| FAM3B | 1.34 | 0.195 |  | PDPK1 | 1.29 | 0.098 |
| FAM3D | 1.65 | **0.046*** |  | PDX1 | 1.10 | 0.527 |
| FBP1 | -1.01 | 0.915 |  | PIK3CA | 1.03 | 0.880 |
| FKBP1B | 1.64 | 0.140 |  | PIK3CB | 1.06 | 0.750 |
| FOXO1 | 1.16 | 0.454 |  | PIK3R1 | 1.56 | 0.404 |
| FOXO4 | -1.08 | 0.686 |  | PRKCA | 1.03 | 0.885 |
| MTOR | 1.76 | 0.307 |  | PRKCD | -1.05 | 0.784 |
| GAB1 | -1.03 | 0.877 |  | PSMD9 | 1.09 | 0.434 |
| GCK | 1.05 | 0.873 |  | PTPN1 | -1.03 | 0.864 |
| GHR | -1.05 | 0.821 |  | PTPRA | 1.10 | 0.111 |
| GHRL | 1.23 | 0.598 |  | PTPRE | 1.02 | 0.899 |
| GPR119 | -1.73 | 0.133 |  | RELA | -1.14 | 0.491 |
| GRB14 | 1.20 | 0.085 |  | RPE65 | 1.22 | 0.515 |
| GRB2 | 1.21 | 0.196 |  | SLC2A2 | -1.20 | 0.527 |
| GSK3A | -1.03 | 0.784 |  | SLC2A4 | 1.16 | 0.193 |
| HNF1A | 1.17 | 0.710 |  | SOCS2 | 1.05 | 0.562 |
| HNF1B | -1.02 | 0.825 |  | TLN1 | 1.04 | 0.898 |
| IDE | -1.04 | 0.859 |  | TLN2 | 2.04 | 0.270 |
| IGF1 | -1.74 | 0.663 |  | TNF | 1.18 | 0.451 |
| IGF1R | -1.07 | 0.738 |  | TRH | 1.23 | 0.598 |
| IGF2 | -1.22 | **0.045*** |  | TSC2 | 1.69 | 0.176 |
| IGF2R | 1.09 | 0.775 |  | UCN3 | 1.14 | 0.829 |

*Note*: **P*<0.05 and ***P*<0.01 compared with LV4(GFP)-NC
